# Exploring the neuroprotective role of ubiquinone by attenuation of oxidative stress and mitochondrial dysfunction in an experimental model of myalgic encephalomyelitis/chronic fatigue stress

**DOI:** 10.1002/alz.085905

**Published:** 2025-01-03

**Authors:** Anushka Vashishth

**Affiliations:** ^1^ BITS Pilani Hyderabad Campus, Hyderabad, Telangana, India; RMIT, Melbourne, VIC Australia

## Abstract

**Background:**

Myalgic encephalomyelitis (ME) or chronic fatigue syndrome (CFS) is categorized as a complicated disorder of extreme fatigue lasting for at least six months without any underlying medical problem and currently has no concrete treatment regimen. This is associated with neurological complications like brain fog, insomnia, psychiatric disturbances and above all neuroinflammation. A chronic forced swim test model of CFS has been established since more than a decade at our laboratory. An important biomarker in CFS is reduced levels of ubiquinone that leads to oxidative stress in neurons, thus impairing neurodevelopment, and ultimately learning and memory.

**Method:**

A 15‐day chronic forced swim daily for 6 minutes induce CFS symptoms in mice. The neurobehavioral studies consist of assessment of locomotor activity, anxiety, motor coordination, cognitive dysfunction in addition to biochemical parameters of catalase, acetylcholinesterase, reduced glutathione, superoxide dismutase, lipid peroxidation and nitrite levels in cortex & hippocampus of mice brain. Histopathological analysis of differences between control and treatment groups was also conducted. Mitochondrial enzyme complex (1‐1V) was done to evaluate mitochondrial dysfunction. An additional *in vitro* study was conducted using PC12 cell lines to study ROS generation and DNA damage following cell viability in an H_2_0_2_ induced oxidative stress model.

**Result:**

Treatment with ubiquinone showed a positive impact by significantly improving the behavioral, biochemical, and cellular parameters of stress after reversing the imbalance between free radicals and anti‐oxidants which is attributed to its anti‐oxidant nature. Histopathological analysis showed significant neuronal loss in diseased group (darkly stained neurons with fragmented or no nucleus and few of the cells were shrunken or sickle shaped and with altered morphology) as compared to naïve group that showed improvement on drug treatment. Results of *in vitro* were consistent with above findings.

**Conclusion:**

As per available literature, this study successfully adds to the potential of ubiquinone as a treatment strategy for neurodegeneration. In an experimental model of CFS for evaluation of neurobehavioral, biochemical, and cellular changes to mitigate neuroinflammation, ubiquinone exhibits its role to be used further in research for severe neurodegenerative disorders like AD and PD. With more research this could lead to a safer and cost‐effective drug target.

